# Personalised circulating tumour DNA assay with large-scale mutation coverage for sensitive minimal residual disease detection in colorectal cancer

**DOI:** 10.1038/s41416-023-02300-3

**Published:** 2023-06-06

**Authors:** Seung-Bum Ryoo, Sunghoon Heo, Yoojoo Lim, Wookjae Lee, Su Han Cho, Jongseong Ahn, Jun-Kyu Kang, Su Yeon Kim, Hwang-Phill Kim, Duhee Bang, Sung-Bum Kang, Chang Sik Yu, Seong Taek Oh, Ji Won Park, Seung-Yong Jeong, Young-Joon Kim, Kyu Joo Park, Sae-Won Han, Tae-You Kim

**Affiliations:** 1 Department of Surgery, Seoul National University Hospital, Seoul, Korea; 2 IMBdx, Seoul, Korea; 3 Department of Chemistry, Yonsei University, Seoul, Korea; 4 Department of Surgery, Seoul National University Bundang Hospital, Seongnam, Korea; 5 Department of Surgery, Asan Medical Center, Seoul, Korea; 6 Department of Surgery, The Catholic University of Korea Uijeongbu St. Mary’s Hospital, Uijeongbu, Korea; 7 Department of Biochemistry, Yonsei University, Seoul, Korea; 8 Department of Internal Medicine, Seoul National University Hospital, Seoul, Korea; 9 Cancer Research Institute, Seoul National University, Seoul, Korea

**Keywords:** Prognostic markers, Colon cancer, Rectal cancer

## Abstract

**Background:**

Postoperative minimal residual disease (MRD) detection using circulating-tumour DNA (ctDNA) requires a highly sensitive analysis platform. We have developed a tumour-informed, hybrid-capture ctDNA sequencing MRD assay.

**Methods:**

Personalised target-capture panels for ctDNA detection were designed using individual variants identified in tumour whole-exome sequencing of each patient. MRD status was determined using ultra-high-depth sequencing data of plasma cell-free DNA. The MRD positivity and its association with clinical outcome were analysed in Stage II or III colorectal cancer (CRC).

**Results:**

In 98 CRC patients, personalised panels for ctDNA sequencing were built from tumour data, including a median of 185 variants per patient. In silico simulation showed that increasing the number of target variants increases MRD detection sensitivity in low fractions (<0.01%). At postoperative 3-week, 21.4% of patients were positive for MRD by ctDNA. Postoperative positive MRD was strongly associated with poor disease-free survival (DFS) (adjusted hazard ratio 8.40, 95% confidence interval 3.49–20.2). Patients with a negative conversion of MRD after adjuvant therapy showed significantly better DFS (*P* < 0.001).

**Conclusion:**

Tumour-informed, hybrid-capture-based ctDNA assay monitoring a large number of patient-specific mutations is a sensitive strategy for MRD detection to predict recurrence in CRC.

## Background

Circulating-tumour DNA (ctDNA), which is found in the bloodstream as a fragmented cell-free DNA (cfDNA), can reflect the genomic landscape of the tumour in a non-invasive manner [1, 2]. It is readily detectable in the majority of metastatic colorectal cancer and ctDNA sequencing can provide a real-time mutational profile of the entire tumour that can assist treatment decisions [3, 4]. Enhanced sensitivity driven by technological advances has also enabled the detection of a trace amount of ctDNA originating from minimal residual disease (MRD) in solid tumours.

The presence of MRD determined using ctDNA analysis has been recently suggested as an important prognostic biomarker. MRD status after surgery was significantly associated with higher recurrence in multiple studies using various assay technics [5–7]. Moreover, a recent prospective clinical trial showed the possibility that ctDNA-guided adjuvant chemotherapy in Stage II colon cancer could reduce unnecessary chemotherapies without compromising survival outcomes [8]. Multiple clinical trials evaluating the clinical utility of ctDNA MRD assessments in predicting prognosis and subsequent stratification of adjuvant chemotherapy intensity are currently ongoing [9, 10].

Detection of ctDNA originating from the MRD after curative treatments in the earlier stage patients requires a more sensitive and robust assay technology compared to the ctDNA analysis of metastatic disease. The amounts of ctDNA from MRD burden below the detection limit of imaging modalities are very scarce, and the strategies used for molecular profiling in metastatic patients cannot capture them in most cases [11]. Various strategies for enhancing the sensitivity, including the adoption of tumour-informed approaches and incorporation of methylation-based markers, are being tested [12, 13]. The tumour-informed approach has been one of the earliest strategies to be used for MRD detection, as it enables deeper sequencing of the most important variants in each patient to maximise the detection sensitivity. Hybridisation capture is a targeted next-generation sequencing method that can support MRD detection by providing the power to target nearly an unlimited number of variants at once.

In this context, we have developed a sensitive tumour-informed, hybrid-capture sequencing-based ctDNA MRD assay (AlphaLiquid®Detect). Here, we report on the results of the performance of the ctDNA MRD assay in relation to the clinical outcomes of Stage II–III CRC patients treated with curative surgery and adjuvant treatment.

## Methods

### AlphaLiquid®Detect workflow for constructing tissue-informed panels

**Fig. 1:**
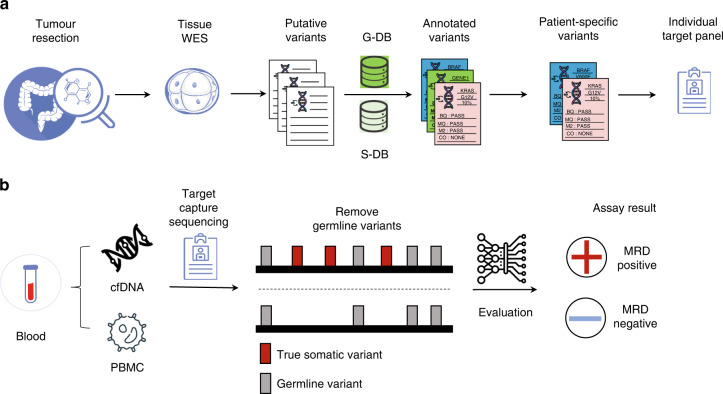
An introduction to the AlphaLiquid®Detect workflow. **a** A workflow of AlphaLiquid®Detect target-capture panel design procedure. After the patient operation, whole-exome sequencing (WES) of tumour tissue is performed. After putative variant calling using tumour tissue only, variants are annotated with germline database (G-DB) and somatic database (S-DB). After the variant annotation, the germline variants are removed using in-house criteria. The remaining variants are further processed to select candidate target variants. These candidate variants include individual-specific germline variants and true somatic variants. These variants are used for individual target-capture panel design. **b** The procedure of AlphaLiquid®Detect assay after the patient’s blood sampling. At the blood sampling, the gDNA from PBMC and cfDNA from the plasma of whole blood are extracted. The extracted DNA samples underwent target-capture sequencing using the individual target-capture panel. Individual-specific germline variants and the clonal hematopoiesis of indeterminate potential (CHIP) variants of cfDNA were excluded using PBMC sequencing data. The remaining variants were further evaluated to test whether the patient is MRD positive or negative.

AlphaLiquid®Detect is a tumour-informed personalised MRD detection assay that examines most of the mutations in the patient’s tumour (Fig. 1). The process starts from the tissue WES data. Adapter sequences and low-quality reads were trimmed using fastp [14]. The trimmed reads were aligned to the reference genome (hg38) using bwa [15] and then further processed following the GATK Best Practices recommendations. Briefly, duplicated reads were marked, and base recalibration was performed to generate analysis-ready BAM files. Variant calling was carried out using Mutect2 [16]. Variants were annotated using multiple databases informing on functional importance or occurrence in general populations: CancerHotspot [17], COSMIC [18], SnpEFF [19], ClinVar [20], dbSNP [21], gnomAD [22] and Korean germline DB [23].

Annotated variants were further processed with an in-house variant selection algorithm (detailed in “Tissue variant selection”). Selected variants were included in the target-capture panel. Effectively, most of the somatic variants found in the WES data with a moderate variant allele frequency were covered in our platform. Per each patient, up to 300 variants were selected. Each tumour-informed panel was constructed using the merged variant sets from four or five patients.

### Tissue variant selection

To select effective tissue markers for monitoring purposes, a series of strategies were implemented: (a) removing potentially germline variants, (b) removing variants in regions prone to sequencing errors or associated with low coverage, (c) prioritising variants with high functional potential, and (d) prioritising variants in well-known oncogenes or with high variant allele frequency.

Specifically, germline variants found with a population allele frequency greater than 0.1% were filtered out. Variants outside of exon regions, overlapping with blacklist regions recognised by large studies such as ENCODE project [24] or in known low mappability regions [25] were also discarded. We then applied an in-house tiering system to prioritise variants. The variants having well-defined clinical evidence based on widely accepted mutation databases such as COSMIC [17] and CancerHotspots [18] were assigned the highest tier. In addition, most of the variants in nearly ~1000 oncogenes accumulated in the in-house database, and the variants with variant allele frequency above 10% were included.

### Blood sample sequencing data pre-processing and detection of minimal residual disease

The sequencing data from blood samples were generated with the unique molecule identifier (UMI) barcode. After removing adapter sequences and low-quality bases using fastp, the raw sequencing data were processed. Specifically, the UMI sequences were extracted from each sequencing read pair and stored in unmapped BAM format. Potential sequencing errors in the UMI sequences were corrected. The reads were aligned onto the reference genome (hg38) using bwa, and the mapped reads were sorted by the genomic coordinate and grouped by each UMI family. The high-quality sequences (HQSs) were then created using the reads in each UMI family group but with a condition suppressing polymerase chain reaction (PCR) or next-generation sequencing errors further, leaving only highly accurate molecular fragments (Supplementary Fig. 1). Finally, the generated HQSs were re-aligned onto the reference genome, generating analysis-ready BAM. Using HQS, the initial variant calls were obtained using VarDict [26], and the variants present in the matched peripheral blood mononuclear cells (PBMC) sample were filtered out, as well as those with low mapping quality and low base quality. Variants residing in the reads with many mismatches were also discarded. The presence of ctDNA was then evaluated using the number of positive variants detected (among the targeted tissue markers). Two or more positive variants were needed to confirm MRD positivity.

### Patient sample processing

Fresh frozen tumour tissues from the included patients were sent to IMBdx, and all subsequent steps from sample DNA extraction were performed at IMBdx. Tumour tissue genomic DNA (gDNA) was extracted from fresh frozen samples using the Maxwell® RSC Tissue DNA Kit (Promega, USA) according to the manufacturer’s instructions. Extracted tumour tissue gDNA was quantified using Qubit dsDNA High Sensitivity Kit (Thermo Fisher Scientific, USA) and fragmented to a target size of 180–220 bp using SureSelectXT HS and XT Low Input enzymatic fragmentation kit (Agilent, USA) according to the manufacturer’s instructions. Then ≤100 ng of sheared DNA was put into library preparation.

Each blood sample was centrifuged with Ficoll solution at 1500×*g* for 15 min, and the plasma and PBMC were transferred from the separated blood. Plasma was separated by centrifugation at 16,000×*g* for 10 min to remove cell debris. In all, 1 mL aliquots of the plasma were placed in Eppendorf tubes and stored at −80 °C before extraction. Plasma and PBMC pellets from enrolled patients were sent to IMBdx, and all subsequent steps from sample DNA extraction were performed at IMBdx.

cfDNA was extracted from 1 to 10 mL of plasma using the Maxwell® RSC cfDNA Plasma Kit (Promega, USA) according to the manufacturer’s instructions. After extraction, cfDNA was quantified using the Cell-free DNA ScreenTape Assay with the 4200 TapeStation Systems (Agilent, USA). Then 2–20 ng of cfDNA was input into library preparation.

gDNA from PBMC pellet was extracted using the Maxwell® RSC Blood DNA Kit (Promega, USA) according to the manufacturer’s instructions, quantified and fragmented in the same manner as gDNA from tumour tissue. Then ≤100 ng of sheared DNA was input into library preparation.

### Library preparation and sequencing

To identify genetic mutations in each patient’s tumour tissue, WES was performed at more than 200X depth coverage on a NovaSeq platform (Illumina, USA) at 2 × 150 bp. An individual-specific variant targeting panel was designed and manufactured using patient-specific genetic mutations detected in WES. For cfDNA of all time points and PBMC gDNA of the first time point, the library preparation is carried out using IMBdx’s AlphaLiquid®Detect platform. After library preparation, hybrid capture is performed using a patient-matched capture panel according to AlphaLiquid®Detect protocol. These target-captured libraries were sequenced on the NovaSeq platform at 2 × 150 bp to achieve the on-target average coverage of 100,000× for cfDNA and 1000× for PBMC.

### Analytical performance validation using mixture samples

To assess the limit of detection of our assay, we evaluated the synthetic mixture samples generated from NA12891 and NA12892 (Coriell Institute, USA) that have known genotypes. We examined the 165 SNP variants specific to NA12892 (i.e., not present in NA12891 nor in the human reference genome) that do not overlap with the repeat regions. The gDNA from the two cell lines were quantified using Qubit dsDNA High Sensitivity Kit (Thermo Fisher Scientific, USA) and fragmented to a target size of 180–220 bp using Covaris S220 sonicator. Then sheared gDNA were quantified using the D1000 ScreenTape Assay with the 4200 TapeStation Systems (Agilent, USA).

The sheared NA12892 gDNA was mixed with the sheared NA12891 gDNA, generating for each mixture proportion ranging in 1%, 0.5%, 0.1%, 0.05%, 0.01%, 0.005%, 0.001% of sheared NA12892 gDNA. Heterozygotes in NA12892 would contribute a single variant copy among two alleles, and alternative homozygotes contribute two variant copies out of two. Thus, we adjusted the weight for the observed allele frequency at each target site considering the genotypes of the cell lines and averaged across all markers. This estimate was compared with the expected (nominal) mixture ratio. Subsequent experiments were performed with triplicate for each mixture. Library preparation and hybrid capture were performed using the IMBdx’s AlphaLiquid®Detect protocol. These synthetic mixture libraries were sequenced on the NovaSeq platform at 2 × 150 bp to achieve the on-target average coverage of 200,000×.

### Patient and samples

We performed a retrospective ctDNA MRD study using a subset of a prospective multi-centre CRC cohort in Korea. The cohort enrolls newly diagnosed, adult patients with Stage II–IV CRC treated with surgery and/or chemotherapy from five university hospitals in Korea. The cohort collects clinical data and banks tumour tissue samples as well as periodic blood samples, to discover multi-omics biomarkers related to CRC prognosis and to establish a large-scale biobank for future clinical research. For this study, we have selected Stage II or III patients treated with standard care surgery with or without adjuvant chemotherapy and/or radiotherapy, that have an adequate amount of archived surgical tissue and blood samples for genomic analysis. For each patient, a surgical tumour sample obtained at the time of surgery, PBMC and cfDNA extracted from the blood sample before surgery, and cfDNA extracted from the blood samples at 3 weeks after surgery (allowed window period of 2–10 weeks) and 1 year after surgery was used. All patients were followed for clinical recurrence for up to 5 years with at least every 6 months for the first 2 years and once a year for the postoperative 3–5 years with plasma carcinoembryonic antigen (CEA) and contrast-enhanced computed tomography scans.

### Statistical analysis

DFS was calculated from the date of surgery to the date of clinically confirmed recurrence or death by any cause and was evaluated with the Kaplan–Meier method. The univariate comparisons of DFS were performed using the log-rank tests, and the multivariate comparisons were performed using the Cox proportional hazard model. The Chi-squared test was used for the comparison of categorical variables, and the Student’s *t* test for the comparison of continuous variables between groups. A *P* value of less than 0.05 was considered statistically significant. The statistical analysis was performed using R (version 4.1.1) software.

The overall sample size was determined to provide a decent number to demonstrate the utility of AlphaLiquid®Detect with 80% power at a type I error rate of 5%. Based on previous MRD studies on Stage II–III colorectal cancer patients [5, 13], we anticipated the positive rate of MRD at postoperative 3-week to be between 15 and 20%, the per-year relapse event rate among the MRD negative patients to be ~5%, and the hazard ratio of recurrence by MRD positivity to be larger than 6. The follow-up time for our study was planned as 3 years or longer, and the per-year dropout rate was anticipated at 3%. Using Schoenfeld’s formula [27] with the most conservative assumptions, the sample size was estimated as 95.

## Results

### Assessment of analytical performance

**Fig. 2:**
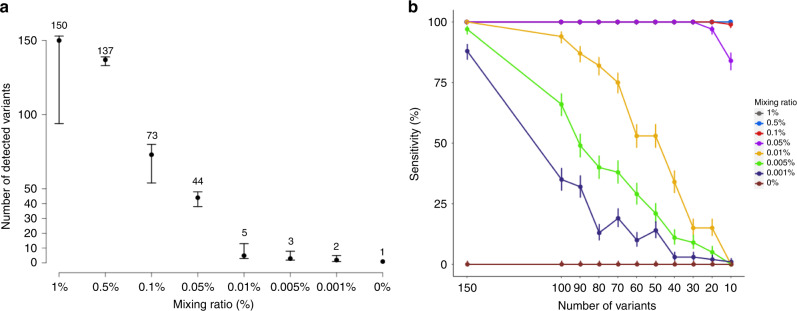
The number of monitoring variants affects the detection power. Synthetic mixture samples were constructed using two cell lines controlling a mixing ratio, which represents a fraction of minimal residual disease in the mixture reflected in the 165 variants specific to only one cell line. At each mixing ratio, three replicates were generated. **a** The number of detected variants among the 165 markers versus the mixing ratio. The median value of three replicates (centre circle) is shown on the top of the bar. The range bar shows the minimum and the maximum number of detected variants at a given mixing ratio. **b** The sensitivity of spike-in DNA by varying the number of monitoring markers with a detection threshold equal to the two. The vertical bar indicates a 90% confidence interval. A random sampling of monitoring markers was performed 100 times without replacement, varying the number of markers.

To examine the preclinical performance of the MRD assay, we created synthetic mixture samples using two cell lines with known single nucleotide variations (NA12891 and NA12892). A target-capture panel was synthesised to include 165 variants specific to NA12892. Forty nanograms of serial dilutions of DNA mixtures of NA12892 in NA12891 ranging from 1 to 0.001% were sequenced using the capture panel. The sequencing depth was 306,754×, and the HQS depth was 5027× on average (Supplementary Table 1). The median number of NA12892 variants detected using the MRD assay dropped from 150 to 2 as the fraction of NA12892 in the mixture decreased from 1 to 0.001% (Fig. 2a).

We have performed an in silico simulation study to examine how the number of variants included in the panel affects the MRD (NA12892 in this experiment) detection sensitivity with a cut-off of two variants for positive MRD. Ten to 150 variants were randomly sub-selected for inclusion in the panel, and the sensitivity of the panels was calculated (Fig. 2b). The simulation test was repeated 100 times with replacement. The simulation result shows that the sensitivity was not affected by the panel size when the mixing ratio is sufficiently high (≥0.5%). In contrast, the sensitivity decreased dramatically with the decrease in the panel size in samples with mixing ratios of <0.01%, which is the range of variant fractions commonly expected with MRD (Fig. 2b). Based on these preclinical data, we have decided to include as many individual variants as possible, up to 300, in the personalised MRD assay and to use a cut-off of two variants for a positive MRD.

### Analysis of ctDNA MRD in clinical samples

Blood and tissue samples from a total of 98 CRC patients were used to examine the diagnostic performance of the MRD assay. Patient characteristics are summarised in Table 1. The median age of the patients was 67 years, and 60 (61.2%) patients were male. In total, 38 (38.8%) of the patients were Stage II, and 60 (61.2%) were Stage III. Patients received adjuvant chemotherapy (83.7%) or radiotherapy (42.9% of rectal cancer) at the discretion of the treating physician. Preoperative, postoperative 3-week and postoperative 1-year blood samples were available from 97, 98, and 94 patients, respectively.

**Table 1:** Patient characteristics.

Characteristics	All patients, *N* = 98 (%)	Postop 3-wk MRD positivity, *N* (%)	*P* value
Age, median (range)	67 (31–87)		
<67	*N* = 48	8 (16.7%)	0.26
≥67	*N* = 50	13 (26.0%)	
Sex			
Male	60 (61.2%)	11 (18.3%)	0.35
Female	38 (38.8%)	10 (26.3%)	
Primary site of disease*			
Right colon	23 (23.5%)	4 (17.4%)	0.64
Left colon	54 (55.1%)	11 (20.4%)	
Rectum	21 (21.4%)	6 (28.6%)	
Stage			
II	38 (38.8%)	2 (5.3%)	0.002
III	60 (61.2%)	19 (31.7%)	
T categories			
T1–3	77 (78.6%)	16 (20.8%)	0.79
T4	21 (21.4%)	5 (23.8%)	
N categories			
N0–1	76 (78.6%)	14 (18.2%)	0.13
N2	21 (21.4%)	7 (33.3%)	
T4 or N2 disease			
No	61 (62.2%)	16.40%	0.12
Yes	37 (37.8%)	29.70%	
Histology			
Adenocarcinoma	93 (94.9%)	19 (20.4%)	0.32^†^
W/D	9 (9.2%)	2 (22.2%)	0.98^‡^
M/D	75 (76.5%)	15 (20.0%)	
P/D	9 (9.2%)	2 (22.2%)	
Mucinous adenocarcinoma	4 (4.1%)	2 (50.0%)	
Undifferentiated carcinoma	1 (1.0%)	0	
Lymphovascular invasion			
No	28 (28.6%)	4 (14.3%)	0.28
Yes	70 (71.4%)	17 (24.3%)	
Microsatellite instability			
MSS/MSI-L	91 (92.9%)	20 (22.0%)	0.63
MSI-H	7 (7.1%)	1 (14.3%)	
Preoperative CEA			
Normal	76 (77.6%)	16 (21.1%)	0.7
Elevated	20 (20.4%)	5 (25.0%)	
NA	2 (2.0%)		

*W/D* well differentiated, *M/D* moderately differentiated, *P/D* poorly differentiated, *MSS* microsatellite stable, *MSI-L* microsatellite instability—low, *MSI-H* microsatellite instability—high, *CEA* carcinoembryonic antigen.

*Right-sided colon cancer: caecum, ascending, hepatic flexure, or transverse colon; left-sided colon cancer: splenic flexure, descending, sigmoid, rectosigmoid colon.

^†^Comparison of MRD positivity between adenocarcinoma, mucinous carcinoma and undifferentiated carcinoma.

^‡^Comparison of MRD positivity by differentiation within adenocarcinoma.

WES was performed with the tumour samples obtained at the time of surgery with a median sequencing depth of 466× (range 275–788). Among the putative somatic variants identified in the tissue WES (median 484, range 281–9235), a median of 185 (range 116–300) variants were used to design individual target-capture panels. Capture probes were synthesised by pooling panels of 4 to 5 patients. Plasma cfDNA was sequenced using the capture probe, and the median sequencing depth was 129,849× (range 16,514–234,362). Genomic DNA from PBMC was also sequenced with the same probe (median depth 3,858X, range 1085–16,791) to remove patient-specific germline SNP. After removing SNP, a median of 66 somatic mutations (range 6–279) per patient were used for MRD monitoring. Most of the mutations (98.4%, 6519/6625) used for monitoring were private mutations found in only one patient (Supplementary Fig. 3).

Among the 97 preoperative blood samples, ctDNA was detectable in 91 (93.8%) samples (median 29 mutations, range 0–234) (Supplementary Figs. 4 and 5). ctDNA mutation was not detected in six patients, and the plasma CEA levels of the six patients were also within the normal ranges. On the other hand, all of the 20 patients having elevated plasma CEA had detectable ctDNA mutations. There was no significant difference in other clinicopathological characteristics, including tumour stage and MSI status according to the preoperative ctDNA positivity (Supplementary Table 2). There was no difference in experimental factors as well, such as cfDNA concentration and sequencing quality (Supplementary Fig. 6).

ctDNA mutations were detected in the postoperative 3-week blood samples from 35.7% of patients (median 2 mutations in positive samples, range 1–72). Among the total of 407 mutations detected at postoperative 3-week, 99.8% were private mutations. Using a cut-off of two mutations for MRD positivity, 21 (21.4%) of the 98 patients were positive for MRD. Patients with Stage III disease were more likely to have a positive MRD than patients with Stage II (*P* = 0.002, Table 1). Only one of the 21 patients with positive MRD had their postoperative plasma CEA elevated at the same time point.

Since the capture probe included individual panels from 4 to 5 patients, we were able to estimate the false positive rate of the assay by analysing the sequenced regions from the other patients’ specific panels included in the capture probe. Using the same MRD cut-off of 2 or more variants for positivity, false positive calls from other patients’ panels were observed in 1.5% of the cross-panel analysis.

### Recurrence according to the ctDNA MRD status

**Fig. 3:**
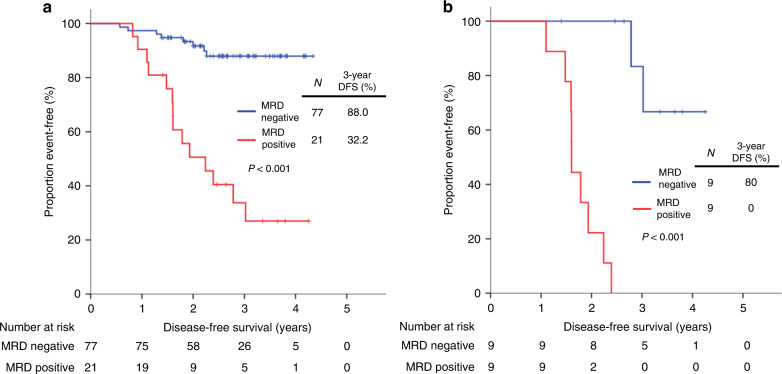
The Kaplan–Meier plots of DFS. **a** According to the postoperative 3-week ctDNA MRD status in all patients, and **b** according to the postoperative 1-year ctDNA MRD status in patients who were MRD positive at postoperative 3-week and subsequently treated with adjuvant therapies.

During a median follow-up duration of 36.3 months (range 16.7–52.1), 22 clinical recurrences were confirmed, and the 3-year disease-free survival (DFS) of the entire cohort was 74.9%. Patients who had positive MRD 3 weeks after surgery showed significantly worse DFS compared with those with negative results (3-year DFS 32.2% vs. 88.0%, respectively; *P* < 0.001, Fig. 3a). In the multivariate analysis adjusting for clinicopathologic prognostic factors, only postoperative 3-week MRD status was significantly associated with DFS (adjusted hazard ratio 8.40, 95% confidence interval 3.49–20.2, Table 2).

**Table 2:** Univariate and multivariate analyses of DFS according to clinicopathological variables and ctDNA MRD status.

	Univariate		Multivariate	
	3 yr DFS	*P* value	HR (95% CI)	*P* value
Age, per-year increase				
<67	78.5%	0.32		
≥67	71.3%			
Tumour site				
Right colon	17.4%	0.59		
Left colon/rectum	22.7%			
Stage				
II	88.4%	0.035*		
III	66.5%			
T categories				
T1–3	77.8%	0.31		
T4	62.3%			
N categories				
N0–1	76.5%	0.33		
N2	68.2%			
T4 or N2 disease				
No	82.2%	0.033*		
Yes	61.7%			
Tumour differentiation				
W/D, M/D, unspecified	75.3%	0.56		
P/D	66.7%			
Microsatellite instability status				
MSS/MSI-L	73.8%	0.61		
MSI-H	85.7%			
Preoperative CEA				
Normal	76.4%	0.13*	2.39	0.061
Elevated	64.2%		(0.96–5.92)	
Postoperative (3-week) ctDNA				
Negative	88.0%	<0.001*	8.40	<0.001
Positive	32.2%		(3.49–20.2)	
Adjuvant chemotherapy/radiotherapy				
No	81.3%	0.81		
Yes	73.8%			

*These variables were included in the multivariate analysis.

Of the 21 patients who were MRD positive at 3 weeks after surgery, 19 patients received adjuvant therapies. The remaining 2 patients who were not treated with adjuvant therapy recurred 9.8 and 13.5 months after surgery, respectively (Supplementary Fig. 3). The postoperative 1-year blood samples were available in 18 of the 19 patients who received adjuvant therapies, excluding one patient who recurred 11 months after surgery. In the 1-year sample, 9 of the 18 patients were converted to negative MRD, whereas 9 patients showed persistent MRD. Patients having a negative conversion of MRD showed significantly longer DFS than those who remained positive despite adjuvant therapies (3-year DFS 80.0% vs. 0%, respectively; *P* < 0.001, Fig. 3b).

The sensitivity and specificity of postoperative 3-week MRD positivity predicting recurrence within 3 years were 61.9% and 83.9%, respectively. In order to assess the impact of the number of mutations monitored in the panel on MRD detection, 4 to 150 mutations were selected based on priority (detail in “Methods”) in individual panels, and the sensitivities of each selection were analysed. The sensitivity predicting recurrence within 3 years decreased from 61.9 to 38.1% as the number of monitoring mutations decreased. The 3-week MRD-positive rate also declined from 21.4 to 9.2%. In silico random subsampling of mutations without prioritisation also showed similar results (Supplementary Fig. 7). These data support the importance of including a large number of mutations in the monitoring panel for sensitive MRD detection.

## Discussion

In this study, we have demonstrated the performance of a tumour-informed, hybrid-capture sequencing-based ctDNA MRD detection assay (AlphaLiquid®Detect) in Stage II or III CRC patients. This approach allows the incorporation of a large number of personal mutations for MRD detection and leads to more sensitive detection, as shown by in silico and simulation studies. The results of the assay in 98 CRC patients showed that MRD detected by the assay using the postoperative 3-week blood samples could predict clinical outcomes better than standard clinicopathologic factors. The clearance or persistence of ctDNA MRD after adjuvant therapies was also significantly related to survival outcomes.

ctDNA, which could represent the tumour cells in a non-invasive manner, is becoming an important means for cancer diagnosis and treatment. Recent advances in related technologies have enabled the detection of MRD from ctDNA. The ctDNA MRD assessed after a radical surgery seems to be able to reflect not only the presence of undetectable minimal distant metastasis but also the quality of surgery and is gaining more attention as a prognostic biomarker. The development of sensitive yet practical assays and clinical studies evaluating the feasibility of ctDNA-led adjuvant therapies are actively ongoing.

Among the various strategies being tested for the detection of MRD by liquid biopsy, a tumour-agnostic approach uses predefined variants known to be associated with cancer. This approach is convenient from an operation point of view and requires less turnaround time. However, flexibility in handling diverse tumour types as well as individual differences, can be limited. Also, distinguishing true mutational signals from errors in the background of large target sites may be practically difficult, given the fact that the allele frequencies of variants in the setting of MRD will frequently be at a very low range (<0.5%). On the other hand, tumour-informed assays detect MRD using a panel built on the basis of the variants specific to a tumour from a given patient. Although tumour-informed assays require a longer turnaround time, they can provide sensitive results and efficient longitudinal monitoring. The tumour-informed selection of the panel can allow the use of a relatively smaller panel for each patient, and focusing on small genomic regions most important for each patient can make ultra-high-depth sequencing possible at a reasonable cost.

Several sensitive MRD detection methods were developed adopting the PCR in conjunction with a tumour-informed strategy [28, 29]. Although the PCR technology can efficiently enrich the target DNA molecules, there are limitations to the technology including the complexity in the selection of PCR-appropriate genomic regions and primer designs, and unbiased amplification between regions. The hybridisation capture methodology can be an alternative to the PCR method. Typically, designing capture probes for target-capture sequencing is not affected by the genomic region, and the size of the panel can be unlimited. Therefore, the hybridisation capture method can target a large number of markers and also offer the possibility of detecting ctDNA existing in low fractions. In addition, the high uniformity capturing efficiency of the hybridisation capture methodology offers non-distorted tumour information of each target loci and unbiased calculation of the tumour burden. The incorporation of UMI at the library preparation step enables the suppression of the stochastic errors generated by various sources of errors. This DNA error-suppressed absolute molecular counting offers accurate ctDNA detection.

There are recent studies showing that better sensitivity could be obtained by increasing the number of monitoring markers [11, 12]. A simulation study using our data showed similar results, showing a continuous decrease in detection sensitivity according to the number of monitoring variants, regardless of variant selection priorities. In MRD settings where there are only trace amounts of total ctDNA in the bloodstream, the amount of each variant captured may often fall under the detection threshold if not completely absent. Increasing the number of markers experimentally led to a decreased chance of false negativity. Our data also showed that the majority (98.4%) of somatic variants found by tumour WES were private mutations, in line with another recent study showing that 99.8% of mutations were specific to each patient [29], as only a small proportion of the mutations that constitute the genomic landscape of each patient’s tumour are from the frequently mutated genes of the tumour type. Therefore, a tumour-informed MRD detection strategy using a large-scale coverage encompassing private mutations can be the most sensitive strategy for MRD detection.

The clinical samples used for this study were collected from all Stage II to III CRC patients undergoing curative-intent treatments. The blood samples were collected in a predetermined amount, at a predetermined time window to eliminate any potential factors that could affect the detection power of the assay. We also ensured that all postoperative 3-week samples were collected before the initiation of adjuvant therapies, for the same reason. The postoperative 3-week ctDNA MRD status was more predictive of recurrence than other well-known clinicopathologic risk factors, similar to the results from other studies [5, 28, 30]. Also, a clear difference in clinical outcomes was observed between the patients who had persistent ctDNA MRD after curative surgery or adjuvant therapies and the patients whose ctDNA MRD was cleared by adjuvant therapies, showing the importance of ctDNA MRD as a sign of persistent disease. Further studies of identifying and intensifying the treatments for patients with persistent disease using the cDNA MRD at an early post-treatment period may improve the overall treatment outcomes.

Although our study showed promising results as another MRD detection platform using liquid biopsy, there are several technical limitations. The hybridisation capture method suffers from G > T transversion artefacts generated by oxidative damage. Recent research showed that the addition of reactive oxygen species scavengers, such as hypotaurine reduced errors introduced by such oxidative damage [31]. Second, the absence of whole-exome sequencing of PBMC at the time of target selection introduces individual-specific germline variants, which were on average 68% of our data. Having a large proportion of germline variants may hinder the effective addition of more somatic variants. In our upgraded version of the panel, the proportion of germline variants was substantially reduced by adopting data from additional germline databases (Supplementary Table 3). Third, our platform relies on the synthesis of target-capture probes which is time-consuming. The average time elapsed for target-capture panel arrival was 8 weeks. The optimisation of the capture probe synthesis procedure will reduce the turnaround time to less than 5 weeks. Of note, the patient pooling strategy adopted in this study was specific only for this retrospective study using archival blood samples. In real clinical settings, individualisation of patient-specific target-capture panel design is possible. Therefore, the turnaround time delay by the patient pooling would not likely occur if we adopted this analysis for actual patients. Lastly, the blood samples were not collected at closer intervals, especially during the postoperative 1-year period, including the immediate post-adjuvant therapy period. A subsequent study of ctDNA MRD detection in a larger prospective cohort designed to overcome such limitations is underway and will provide more evidence for future clinical applications.

## Supplementary information

Supporting information

REMARK checklist

## Data Availability

All data generated or analysed during this study are included in this article and the supplementary information files.
