# Supplementary material for: Personalised circulating tumour DNA assay with large-scale mutation coverage for sensitive minimal residual disease detection in colorectal cancer
Source: Br J Cancer. 2023 Jun 6;129(2):374–81. doi: 10.1038/s41416-023-02300-3 (PMC10338477; doi:10.1038/s41416-023-02300-3)
Supplement: Supplementary file 1 — Supporting information [file 41416_2023_2300_MOESM1_ESM.docx]

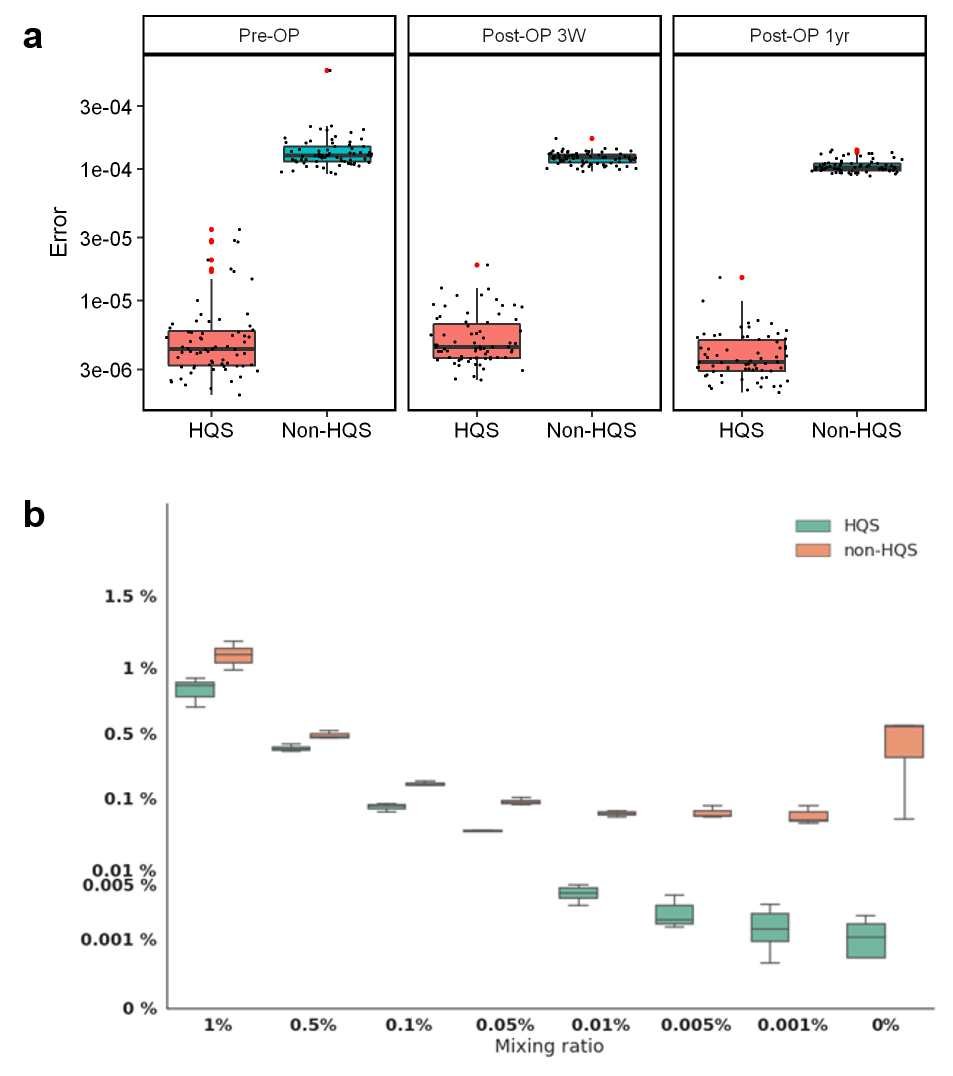


**Supplementary Figure 1. The high-quality sequence (HQS) achieves lower error rate than non-HQSs**. Because our HQS technology utilizes both UMI technology and improved adapter ligation chemistry, the ability to suppress the stochastic error is improved. **(a)** The global error rates estimated from the sequencing data of clinical samples. HQS demonstrated lower error rates compared to non-HQS nearly in the order of 100-fold. **(b)** The estimated mixing proportion in the cell line mixture data. Even when the true mixing ratio is below 0.05%, the detection limit by the conventional error suppression technology (based on non-HQS) only reaches roughly 0.1%, while HQS achieves a much lower detection limit as low as 0.001%.


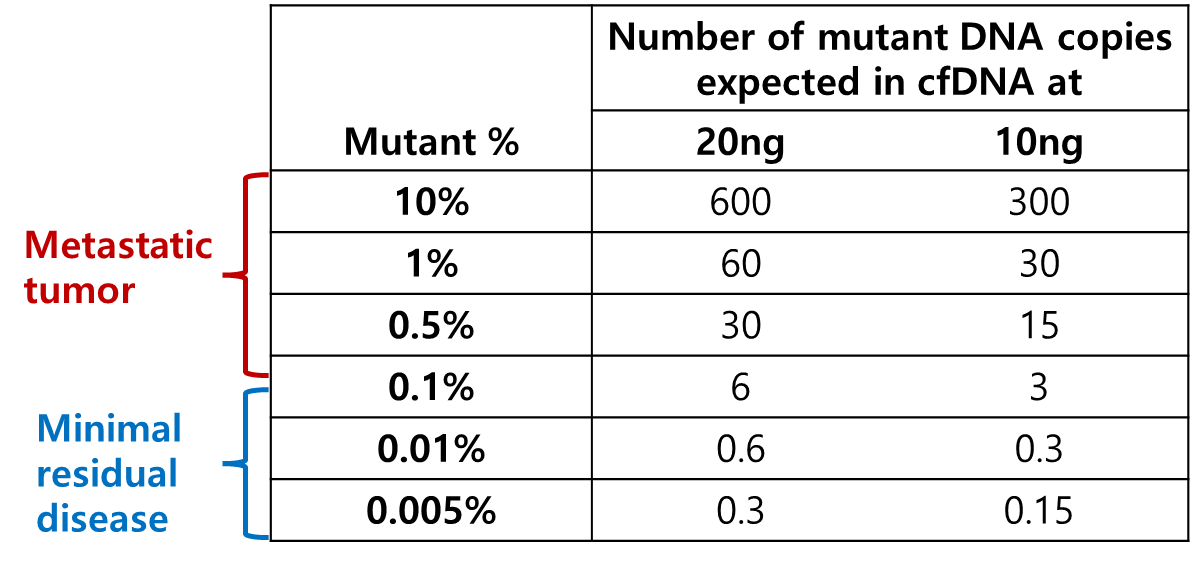


**Supplementary Figure 2.** **The scarce amount of ctDNA expected with MRD burden calls for a large-size personalized panel.** The number of mutant DNA copies in blood dramatically decreases with the decrease in the mutant fraction. In contrast to metastatic condition where observed variant allele fractions (VAF) are easily > 0.5%, the expected mutant fragments in plasma are near or less than 1 copy in MRD condition (VAF is ~0.01% or below). With 20ng of cfDNA, the expected total and mutant DNA copies are 6,000 and 0.6, and with 10ng of cfDNA they are reduced to 3,000 and 0.3.


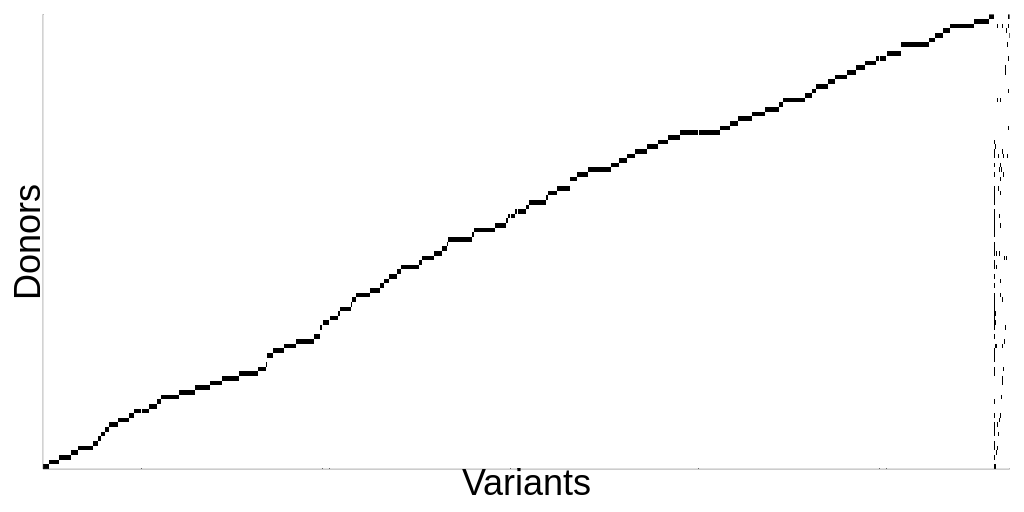


**Supplementary Figure 3. Majority of the monitoring variants derived from the tumor data are private mutations.** Each row denotes the donor tested and each column denotes a monitoring variant (found from any of the 98 clinical samples). For each donor, the associated monitoring variant is marked with a black vertical segment. Private mutations (98.4%, 6,519/6,625) are shown first (left side), and the shared variants among any of the patients are shown on the right side.


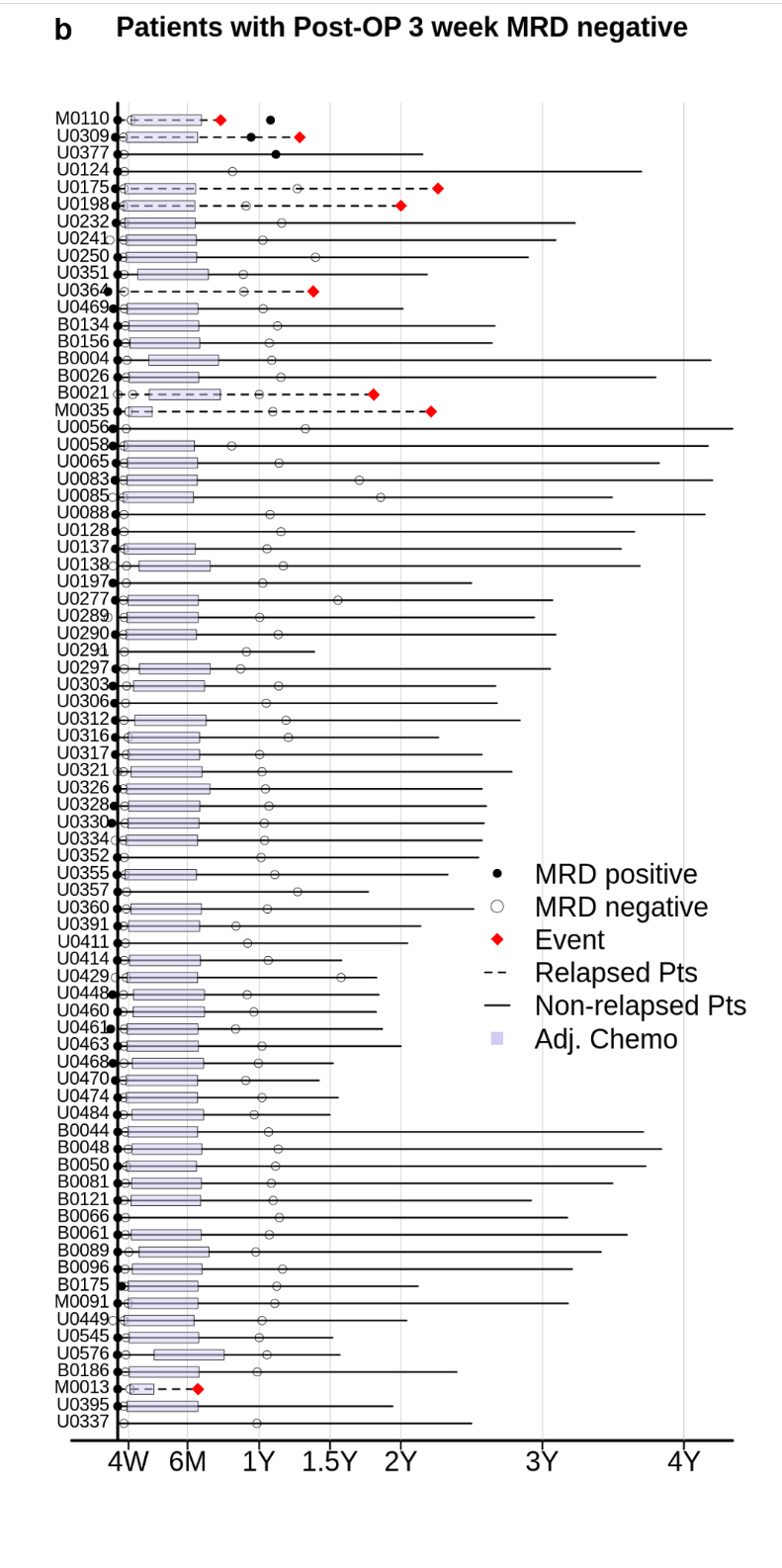

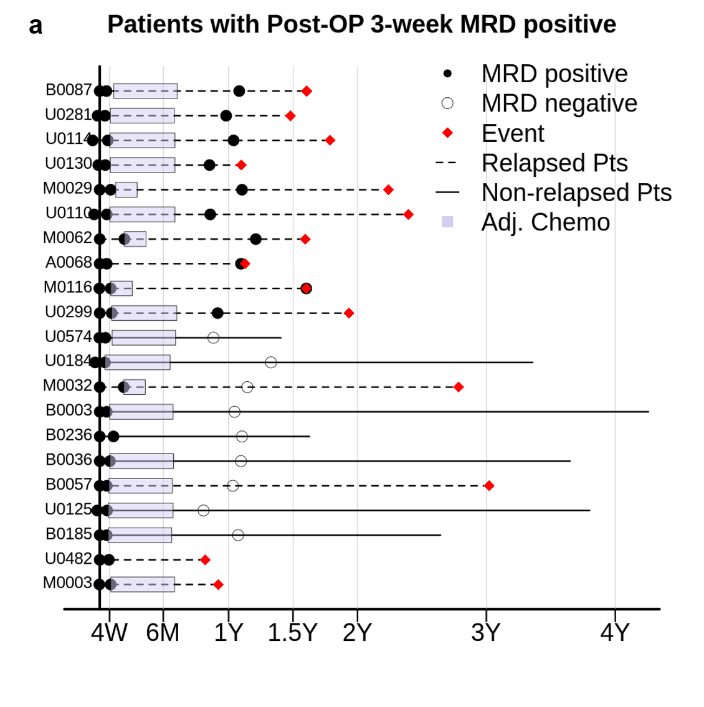


**Supplementary Figure 4. Swimmer plots of the analyzed patients. (a)** An overview of patients who were MRD positive and **(b)** negative at the postoperative 3-week time point. Filled black circle denotes a MRD positive result at the given time point, and hollow circle denotes a MRD negative. Relapsed patients are indicated with a dashed line, and a red diamond denotes the recurrence event. Non-relapsed patients are indicated with a solid line. For each patient, the adjuvant chemotherapy period is marked with a light purple box.


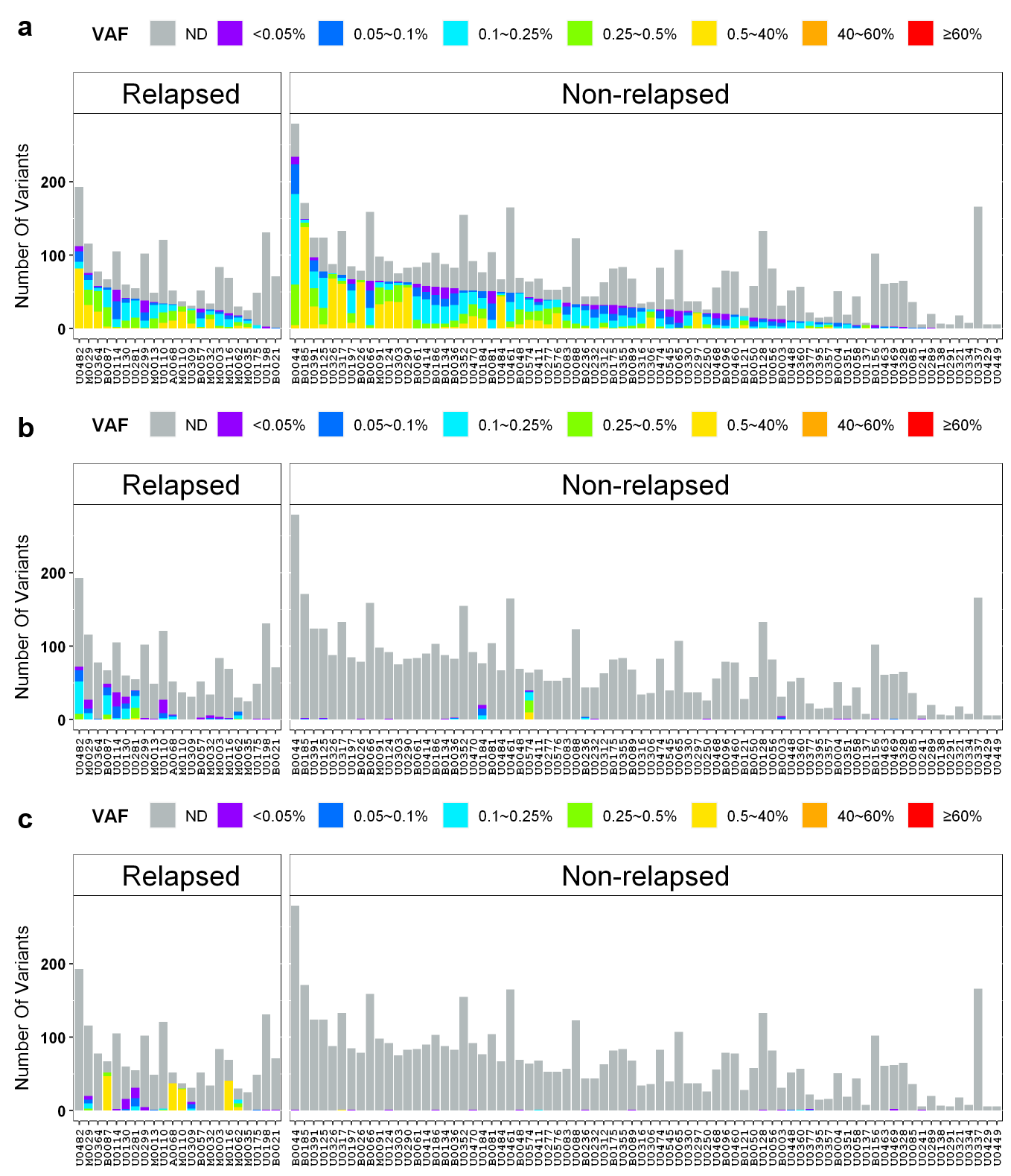


**Supplementary Figure 5. The number of monitoring variants in each patient and its breakdown by the observed VAF range.** For each patient, the numbers of variants in each VAF category (not-detected, <0.05%, 0.05-0.1%, 0.1-0.25%, 0.25-0.5%, 0.5-40%, 40-60%, or 60%) are indicated as a stacked vertical bar where color refers to a specific VAF range. Within each relapsed (left) and non-relapsed (right) group, patients are ordered by the number of detected mutations in the preoperative sample. For the matched patients, the results for the preoperative, postoperative 3-week, postoperative 1year or later samples are shown in the upper, middle, and the lower panel, respectively. **(a)** Preoperative blood sample result shows that tumor variants were observed from majority of the patients. Most of the detected somatic mutations have a variant allele frequency (VAF) > 0.1%. **(b)** For the postoperative 3-week samples, mutations were not detected in majority of the patients and even if identified, the observed VAF is often less than 0.1%. **(c)** Postoperative 1-year or later samples showed that the detected mutations from the relapsed patients often have a relatively high VAF (> 0.5%).


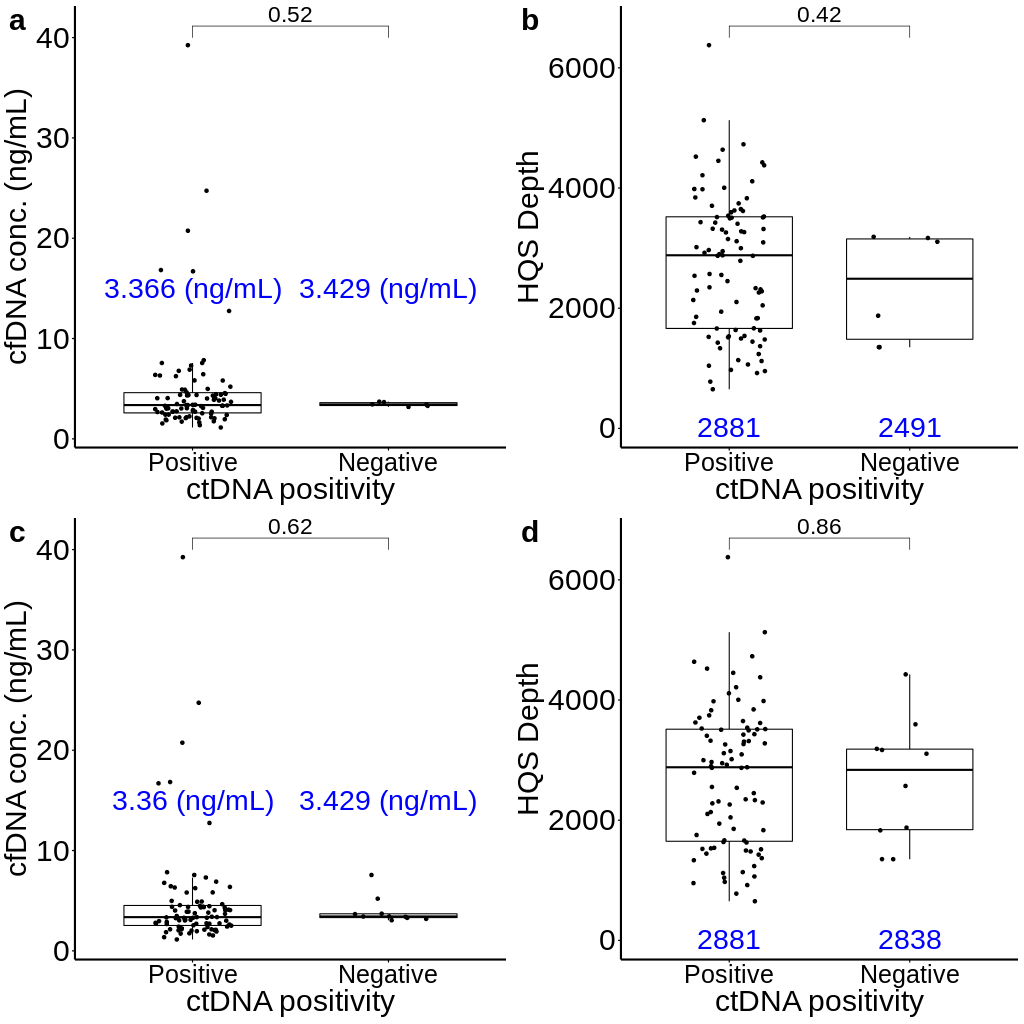


**Supplementary Figure 6. The robustness of AlphaLiquid®Detect assay. (a)** The cfDNA concentration of the preoperative samples used (n=97) showed there is no significant difference between ctDNA positive and negative groups when the detection threshold is 1. **(b)** The HQS depth was not significantly different between ctDNA positive and negative groups when the detection threshold is 1. **(c)** cfDNA concentration difference with threshold 2 and **(d)** HQS depth difference with threshold 2 was not significant. P-value was calculated with Student’s t-test.


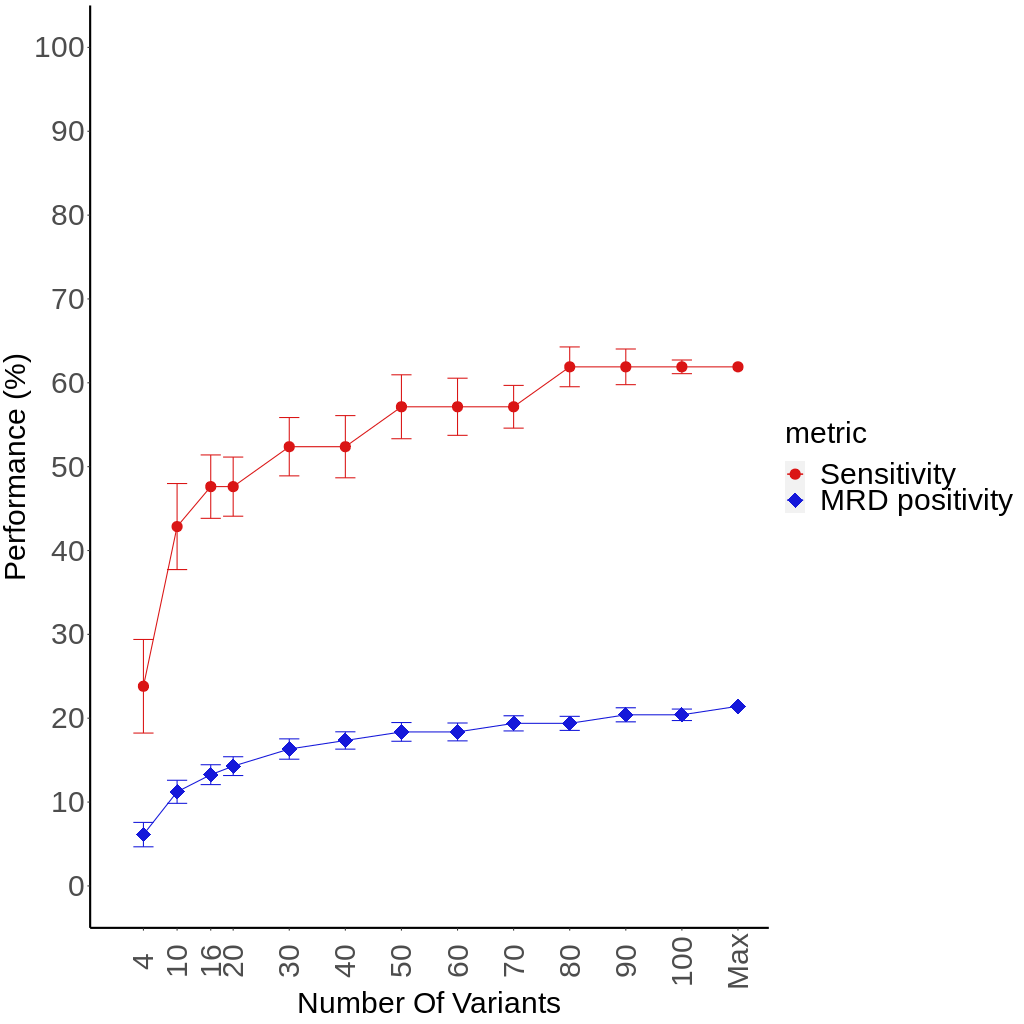


**Supplementary Figure 7.** **The number of variants affects the performance of MRD detection assay.** At each given number of variants, somatic variants were randomly sampled hundred times without replacement. The increment of the markers results in the increment in clinical sensitivity. Moreover, the MRD positivity gradually increases as the number of monitoring variants increases namely a higher probability of MRD detection. Each point denotes the median of the 100 trials. The error bar here indicates the standard error.

**Supplementary Table 1. The sequencing statistics of the analytical validation samples**

| **Sample** | **Input** | **Replicate** | **RawBases** | **OntargetRatio** | **SequencingDepth** | **MoleculeDepth** |
| --- | --- | --- | --- | --- | --- | --- |
| NA12892 VAF 1% | 40 | rep1 | 58,081,502,512 | 62.01 | 316,761 | 4,997 |
| NA12892 VAF 1% | 40 | rep2 | 52,965,943,840 | 61.11 | 284,757 | 4,747 |
| NA12892 VAF 1% | 40 | rep3 | 56,728,527,412 | 60.88 | 304,164 | 2,389 |
| NA12892 VAF 0.5% | 40 | rep1 | 46,946,529,670 | 64.35 | 261,046 | 4,627 |
| NA12892 VAF 0.5% | 40 | rep2 | 49,788,164,846 | 61.97 | 271,118 | 4,787 |
| NA12892 VAF 0.5% | 40 | rep3 | 34,921,461,960 | 61.72 | 200,138 | 6,060 |
| NA12892 VAF 0.1% | 40 | rep1 | 53,874,797,438 | 63.36 | 294,805 | 4,222 |
| NA12892 VAF 0.1% | 40 | rep2 | 55,263,196,534 | 61.98 | 306,205 | 5,074 |
| NA12892 VAF 0.1% | 40 | rep3 | 55,383,340,590 | 63.84 | 323,416 | 6,274 |
| NA12892 VAF 0.05% | 40 | rep1 | 49,177,346,290 | 61.61 | 282,552 | 5,660 |
| NA12892 VAF 0.05% | 40 | rep2 | 61,487,679,274 | 62.71 | 364,700 | 5,828 |
| NA12892 VAF 0.05% | 40 | rep3 | 49,408,934,688 | 54.57 | 247,726 | 6,518 |
| NA12892 VAF 0.01% | 40 | rep1 | 48,516,991,580 | 59.84 | 253,168 | 4,353 |
| NA12892 VAF 0.01% | 40 | rep2 | 70,718,393,230 | 61.39 | 375,053 | 4,688 |
| NA12892 VAF 0.01% | 40 | rep3 | 50,404,185,956 | 62.3 | 289,620 | 5,971 |
| NA12892 VAF 0.005% | 40 | rep1 | 57,634,438,926 | 63.27 | 319,587 | 4,626 |
| NA12892 VAF 0.005% | 40 | rep2 | 61,160,293,456 | 63.74 | 340,866 | 4,681 |
| NA12892 VAF 0.005% | 40 | rep3 | 47,611,845,032 | 61.96 | 274,317 | 6,172 |
| NA12892 VAF 0.001% | 40 | rep1 | 58,010,104,578 | 64.75 | 323,917 | 4,304 |
| NA12892 VAF 0.001% | 40 | rep2 | 149,991,000,000 | 62.65 | 808,589 | 4,549 |
| NA12892 VAF 0.001% | 40 | rep3 | 35,147,280,044 | 64.3 | 205,695 | 5,566 |
| NA12891 WT (NA12892 0%) | 40 | rep1 | 49,049,727,734 | 34.37 | 155,749 | 5,808 |
| NA12891 WT (NA12892 0%) | 40 | rep2 | 54,624,329,728 | 55.23 | 274,063 | 5,817 |
| NA12891 WT (NA12892 0%) | 40 | rep3 | 48,464,094,468 | 63.9 | 284,104 | 2,947 |

**Supplementary Table 2. Clinicopathologic factor analysis for MRD**

|  | | Cut-off = 1 | | | Cut-off = 2 | | |
| --- | --- | --- | --- | --- | --- | --- | --- |
|  |  | ctDNA (+) | ctDNA (-) |  | ctDNA (+) | ctDNA (-) |  |
| Stage | II | 33 (36.3%) | 2 (33%) | p=1 | 33 (37%) | 4 (33%) | p=1 |
|  | III | 58 (63.7%) | 4 (67%) |  | 54 (63%) | 6 (67%) |  |
| MSI | MSI-H | 6 (6.6%) | 0 (0%) | p=1 | 6 (6.9%) | 0 (0%) | p=0.87 |
|  | MSS/MSI-L | 85 (93.4%) | 6 (100%) |  | 81 (93.1%) | 10 (100%) |  |

**Supplementary Table 3. Comparison of the portion of germline variants after the algorithm improvement.**

| Range (Median, min- max; %) | Before optimization  (this study) | After optimization |
| --- | --- | --- |
|  | 68 (44.6-95.5) | 49 (26.1-93) |
